# Evaluation of National Surgical Practice for Lateral Lymph Nodes in Rectal Cancer in an Untrained Setting

**DOI:** 10.1245/s10434-023-13460-0

**Published:** 2023-06-20

**Authors:** Tania C. Sluckin, Sanne-Marije J. A. Hazen, Karin Horsthuis, Regina G. H. Beets-Tan, Arend G. J. Aalbers, Geerard L. Beets, Evert-Jan G. Boerma, Jaap Borstlap, Vivian van Breest Smallenburg, Jacobus W. A. Burger, Rogier M. P. H. Crolla, Alette W. Daniëls-Gooszen, Paul H. P. Davids, Michalda S. Dunker, Hans F. J. Fabry, Edgar J. B. Furnée, Renza A. H. van Gils, Robbert J. de Haas, Stefan Hoogendoorn, Sebastiaan van Koeverden, Fleur I. de Korte, Steven J. Oosterling, Koen C. M. J. Peeters, Lisanne A. E. Posma, Bareld B. Pultrum, Joost Rothbarth, Harm J. T. Rutten, Renske A. Schasfoort, Wilhelmina H. Schreurs, Petra C. G. Simons, Anke B. Smits, Aaldert K. Talsma, G. Y. Mireille The, Fiek van Tilborg, Jurriaan B. Tuynman, Inge J. S. Vanhooymissen, Anthony W. H. van de Ven, Emiel G. G. Verdaasdonk, Maarten Vermaas, Roy F. A. Vliegen, F. Jeroen Vogelaar, Marianne de Vries, Joy C. Vroemen, Sebastiaan T. van Vugt, Marinke Westerterp, Henderik L. van Westreenen, Johannes H. W. de Wilt, Edwin S. van der Zaag, David D. E. Zimmerman, Corrie A. M. Marijnen, Pieter J. Tanis, Miranda Kusters, Dutch Snapshot Research Group, Susanna M. van Aalten, Femke J. Amelung, Marjolein Ankersmit, Imogeen E. Antonisse, Jesse F. Ashruf, Tjeerd S. Aukema, Henk Avenarius, Renu R. Bahadoer, Frans C. H. Bakers, Ilsalien S. Bakker, Fleur Bangert, Renée M. Barendse, Heleen M. D. Beekhuis, Willem A. Bemelman, Maaike Berbée, Shira H. de Bie, Robert H. C. Bisschops, Robin D. Blok, Liselotte W. van Bockel, Anniek H. Boer, Frank C.den Boer, Leonora S. F. Boogerd, Wernard A. A. Borstlap, Johanna E. Bouwman, Sicco J. Braak, Manon N. G. J. A. Braat, Jennifer Bradshaw, Amarins T. A. Brandsma, Wim T. van den Broek, Sjirk W. van der Burg, Thijs A. Burghgraef, David W. G. ten Cate, Heleen M. Ceha, Jeltsje S. Cnossen, Robert R. J. Coebergh van den Braak, Esther C. J. Consten, Maaike Corver, Sam Curutchet, Emmelie N. Dekker, Jan Willem T. Dekker, Ahmet Demirkiran, Tyche Derksen, Arjen L. Diederik, Anne M. Dinaux, Kemal Dogan, Ilse M. van Dop, Kitty E. Droogh-de Greve, Hanneke M. H. Duijsens, Johan Duyck, Eino B. van Duyn, Laurentine S. E. van Egdom, Bram Eijlers, Youssef El-Massoudi, Saskia van Elderen, Anouk M. L. H. Emmen, Marc Engelbrecht, Anne C. van Erp, Jeroen A. van Essen, Thomas Fassaert, Eline A. Feitsma, Shirin S. Feshtali, Bas Frietman, Anne M. van Geel, Elisabeth D. Geijsen, Anna A. W. van Geloven, Michael F. Gerhards, Hugo Gielkens, Lucas Goense, Marc J. P. M. Govaert, Wilhelmina M. U. van Grevenstein, E. Joline de Groof, Irene de Groot, Nadia A. G. Hakkenbrak, Mariska D.den Hartogh, Vera Heesink, Joost T. Heikens, Ellen M. Hendriksen, Sjoerd van den Hoek, Erik J. R. J. van der Hoeven, Christiaan Hoff, Anna Hogewoning, Cornelis R. C. Hogewoning, Roel Hompes, Francois van Hoorn, René L. van der Hul, Rieke van Hulst, Farshad Imani, Bas Inberg, Martijn P. W. Intven, Pedro Janssen, Chris E. J. de Jong, Jacoline Jonkers, Daniela Jou-Valencia, Bas Keizers, Stijn H. J. Ketelaers, Eva Knöps, Sylvia Kok, Stephanie E. M. Kolderman, Robert T. J. Kortekaas, Julie C. Korving, Ingrid M. Koster, Jasenko Krdzalic, Pepijn Krielen, Leonard F. Kroese, Eveline J. T. Krul, Derk H. H. Lahuis, Bas Lamme, An A. G. van Landeghem, Jeroen W. A. Leijtens, Mathilde M. Leseman-Hoogenboom, Manou S. de Lijster, Martijn S. Marsman, Milou.H. Martens, Ilse Masselink, Wout van der Meij, Philip Meijnen, Jarno Melenhorst, Dietrich J. L. de Mey, Julia Moelker-Galuzina, Linda Morsink, Erik J. Mulder, Karin Muller, Gijsbert D. Musters, Joost Nederend, Peter A. Neijenhuis, Lindsey C. F. de Nes, M. Nielen, Jan B. J. van den Nieuwboer, Jonanne F. Nieuwenhuis, Joost Nonner, Bo J. Noordman, Stefi Nordkamp, Pim B. Olthof, Daan Ootes, Vera Oppedijk, Pieter Ott, Ida Paulusma, Ilona T. A. Pereboom, Jan Peringa, Zoë Pironet, Joost D. J. Plate, Fatih Polat, Ingrid G. M. Poodt, Jeroen F. Prette, Seyed M. Qaderi, Jan M. van Rees, Rutger-Jan Renger, Anouk J. M. Rombouts, Lodewijk J. Roosen, Ellen A. Roskott-ten Brinke, Dennis B. Rouw, Tom Rozema, Heidi Rütten, Marit E. van der Sande, Boudewijn E. Schaafsma, Merel M. Scheurkogel, Arjan P. Schouten van der Velden, Puck M. E. Schuivens, Colin Sietses, Marjan J. Slob, Gerrit D. Slooter, Martsje van der Sluis, Bo P. Smalbroek, Ernst J. Spillenaar-Bilgen, Patty H. Spruit, Tanja C. Stam, Sofieke J. D. Temmink, Jeroen A. W. Tielbeek, Aukje A. J. M. van Tilborg, Dorothée van Trier, Maxime J. M. van der Valk, G. Boudewijn C. Vasbinder, Cornelis J. Veeken, Laura A. Velema, Wouter M. Verduin, Tim Verhagen, Paul M. Verheijen, An-Sofie E. Verrijssen, Anna V. D. Verschuur, Harmke Verwoerd-van Schaik, Sophie Voets, Clementine L. A. Vogelij, Johanna Vos-Westerman, Johannes A. Wegdam, Bob J. van Wely, Paul P. van Westerveld, Allard G. Wijma, Bart W. K. de Wit, Fennie Wit, Karlijn Woensdregt, Victor van Woerden, Floor S. W. van der Wolf, Sander van der Wolk, Johannes M. Wybenga, Bobby Zamaray, Herman J. A. Zandvoort, Dennis van der Zee, Annette Zeilstra, Kang J. Zheng, Marcel Zorgdrager

**Affiliations:** 1 Department of Surgery, Amsterdam UMC, Location Vrije Universiteit Amsterdam, Amsterdam, the Netherlands; 2 Cancer Center Amsterdam, Treatment and Quality of Life, Amsterdam, the Netherlands; 3 Cancer Center Amsterdam, Imaging and Biomarkers, Amsterdam, the Netherlands; 4 Department of Radiology and Nuclear Medicine, Amsterdam UMC Location Vrije Universiteit Amsterdam, Amsterdam, the Netherlands; 5 Department of Radiology, the Netherlands Cancer Institute, Amsterdam, the Netherlands; 6 GROW School for Oncology and Reproduction, University of Maastricht, Maastricht, the Netherlands; 7 Department of Radiology, Odense University Hospital, Odense, Denmark; 8 Department of Clinical Research, University of Southern Denmark, Odense, Denmark; 9 Department of Surgery, The Netherlands Cancer Institute, Amsterdam, the Netherlands; 10 Department of Surgery, Zuyderland Medical Center, Sittard-Geleen, the Netherlands; 11 Department of Radiology, Treant Zorggroep, Hoogeveen, the Netherlands; 12 Department of Radiology, Jeroen Bosch Hospital, ’s-Hertogenbosch, the Netherlands; 13 Department of Surgery, Catharina Hospital, Eindhoven, the Netherlands; 14 Department of Surgery, Amphia Hospital, Breda, the Netherlands; 15 Department of Radiology, Catharina Hospital, Eindhoven, the Netherlands; 16 Department of Surgery, Diakonessenhuis, Utrecht, the Netherlands; 17 Department of Surgery, Northwest Clinics, NWZ Alkmaar, Alkmaar, the Netherlands; 18 Department of Surgery, Bravis Hospital, Roosendaal, the Netherlands; 19 Division of Abdominal Surgery, Department of Surgery, University of Groningen, University Medical Center Groningen, Groningen, the Netherlands; 20 Department of Radiology, Erasmus MC, Rotterdam, the Netherlands; 21 Department of Radiology, University of Groningen, University Medical Center Groningen, Groningen, the Netherlands; 22 Department of Radiology, Isala, Zwolle, the Netherlands; 23 Department of Radiology, Radboud University Medical Center, Nijmegen, the Netherlands; 24 Department of Radiology, Haaglanden Medical Centre, The Hague, the Netherlands; 25 Department of Surgery, Spaarne Gasthuis, Haarlem, the Netherlands; 26 Department of Surgery, Leiden University Medical Center, Leiden, the Netherlands; 27 Department of Surgery, Slingeland Hospital, Doetinchem, the Netherlands; 28 Department of Surgery, Martini Hospital, Groningen, the Netherlands; 29 Department of Surgical Oncology and Gastrointestinal Surgery, Rotterdam, the Netherlands; 30 Department of Surgery, Treant Zorggroep, Hoogeveen, the Netherlands; 31 Department of Radiology, VieCuri Medical Centre, Venlo, the Netherlands; 32 Department of Surgery, St. Antonius Hospital, Nieuwegein, the Netherlands; 33 Department of Surgery, Deventer Hospital, Deventer, the Netherlands; 34 Department of Radiology, Bravis Hospital, Roosendaal, the Netherlands; 35 Department of Radiology, Elisabeth-TweeSteden Hospital, Tilburg, the Netherlands; 36 Department of Surgery, Flevoziekenhuis, Almere, the Netherlands; 37 Department of Surgery, Jeroen Bosch Hospital, ‘s-Hertogenbosch, the Netherlands; 38 Department of Surgery, IJsselland Hospital, Capelle aan den IJssel, the Netherlands; 39 Department of Radiology, Zuyderland Medical Center, Sittard-Geleen, the Netherlands; 40 Department of Surgery, VieCuri Medical Centre, Venlo, the Netherlands; 41 Department of Radiology, Flevoziekenhuis, Almere, the Netherlands; 42 Department of Surgery, Wilhelmina Assen Hospital, Assen, the Netherlands; 43 Department of Surgery, Haaglanden Medical Centre, The Hague, the Netherlands; 44 Department of Surgery, Isala, Zwolle, the Netherlands; 45 Department of Surgery, Radboud University Medical Center, Nijmegen, the Netherlands; 46 Department of Surgery, Gelre Hospital, Apeldoorn, the Netherlands; 47 Department of Surgery, Elisabeth-TweeSteden Hospital, Tilburg, the Netherlands; 48 Department of Radiation Oncology, Netherlands Cancer Institute, Amsterdam, the Netherlands; 49 Department of Radiation Oncology, Leiden University Medical Center, Leiden, the Netherlands; 50 Department of Surgery, Amsterdam UMC Location University of Amsterdam, Amsterdam, the Netherlands

## Abstract

**Background:**

Involved lateral lymph nodes (LLNs) have been associated with increased local recurrence (LR) and ipsi-lateral LR (LLR) rates. However, consensus regarding the indication and type of surgical treatment for suspicious LLNs is lacking. This study evaluated the surgical treatment of LLNs in an untrained setting at a national level.

**Methods:**

Patients who underwent additional LLN surgery were selected from a national cross-sectional cohort study regarding patients undergoing rectal cancer surgery in 69 Dutch hospitals in 2016. LLN surgery consisted of either ‘node-picking’ (the removal of an individual LLN) or ‘partial regional node dissection’ (PRND; an incomplete resection of the LLN area). For all patients with primarily enlarged (≥7 mm) LLNs, those undergoing rectal surgery with an additional LLN procedure were compared to those  undergoing only rectal resection.

**Results:**

Out of 3057 patients, 64 underwent additional LLN surgery, with 4-year LR and LLR rates of 26% and 15%, respectively. Forty-eight patients (75%) had enlarged LLNs, with corresponding recurrence rates of 26% and 19%, respectively. Node-picking (*n* = 40) resulted in a 20% 4-year LLR, and a 14% LLR after PRND (*n* = 8; *p* = 0.677). Multivariable analysis of 158 patients with enlarged LLNs undergoing additional LLN surgery (*n* = 48) or rectal resection alone (*n* = 110) showed no significant association of LLN surgery with 4-year LR or LLR, but suggested higher recurrence risks after LLN surgery (LR: hazard ratio [HR] 1.5, 95% confidence interval [CI] 0.7–3.2, *p* = 0.264; LLR: HR 1.9, 95% CI 0.2–2.5, *p* = 0.874).

**Conclusion:**

Evaluation of Dutch practice in 2016 revealed that approximately one-third of patients with primarily enlarged LLNs underwent surgical treatment, mostly consisting of node-picking. Recurrence rates were not significantly affected by LLN surgery, but did suggest worse outcomes. Outcomes of LLN surgery after adequate training requires further research.

Lateral lymph nodes (LLNs) are located outside the mesorectal fascia in the internal iliac and/or obturator (lateral) compartments, and are therefore not removed during standard total mesorectal excision (TME) surgery for rectal cancer patients. LLNs seem to play an important role in the etiology of (lateral) local recurrences ([L]LR).^1–3^ Recent studies have established short-axis diameter to be a main predictor of LLN involvement; LLNs with a short-axis diameter of ≥7 mm were associated with a 5-year LLR rate of up to 19.5%.^4–7^ This warrants improvement, but there is an ongoing international debate regarding the optimal treatment of LLNs.

Surgical treatment of LLNs can be either prophylactic or therapeutic, the latter mostly following neoadjuvant (chemo)radiotherapy ([C]RT). Japanese centers have traditionally performed a prophylactic LLN dissection (LLND) for all cases of advanced rectal cancer.^8–11^ Formal LLND follows anatomical borders in order to remove all lymphatic tissue from within the lateral compartments. The associated risk of bleeding and/or nerve damage is an important reason why Western surgeons have been reluctant to perform LLND. Instead, they rely predominantly on neoadjuvant CRT to sterilize the lateral compartments. However, neither strategy is always sufficient.^12–14^

Recent research has focused on the selective LLND for ‘high-risk’ patients, such as those with primarily enlarged LLNs (≥7 mm)^7^ or persistently enlarged LLNs after CRT.^6^ Using this method instead of prophylactic LLND would potentially reduce the total number of LLNDs, thereby optimizing the harm–benefit ratio. Several studies show the merits of this approach, with reassuring long-term recurrence rates of around 6% or lower.^5,6^ An entirely different approach is the singular removal of only the ‘suspicious’ LLN, often referred to as ‘node-picking’. Only two studies with very small samples have commented on node-picking and results suggest that this approach is inadequate in reducing the LLR rates sufficiently, with 5-year LLR rates up to 51%.^7,15^ Interestingly, in a recent survey of 62 Dutch colorectal surgeons, 16/62 (26%) responded that node-picking was their routine practice for suspicious LLNs, while 27/62 (44%) had performed an LLND at least once in the past 5 years. When asked what the ideal treatment of suspicious LLNs should be, 12/62 (19%) answered node-picking, 44/62 (71%) answered LLND, and 6 (10%) would not perform surgery at all.^16^

This study aimed to evaluate the application of surgical procedures for suspicious LLNs in an untrained setting at a national level, and to compare associated recurrence rates among patients with enlarged LLNs who underwent TME surgery with or without any type of additional LLN surgery.

## Methods

Patients were selected from a national, cross-sectional cohort study of all patients operated on for rectal cancer in the Netherlands between 1 January and 31 December 2016. The short-term oncological outcomes registered for these patients in the Dutch ColoRectal Audit (DCRA) formed the basis of this study. These data were elaborated to include additional diagnostic, therapeutic, and long-term oncological outcomes. Local research teams were formed in each participating center to collect data for their patients, and included a surgeon, surgical residents, radiologist, and radiation oncologist. Data were stored anonymously in a dataset managed by Medical Research Data Management (MRDM). More details can be found in Appendix 1.

While this Snapshot study entails three sections, only the first two are relevant here. During Part one, the surgical team gathered baseline, procedural, and long-term outcomes. Surgical reports were provided for patients who underwent an additional procedure for LLNs and these reports were later reviewed by the central researchers to classify the procedure that was performed. LLN surgery was classified as either formal LLND with complete removal of the internal and obturator compartments, partial regional node dissection (PRND), or node-picking in the case of removal of a single LLN. During Part two, magnetic resonance imaging (MRI) scans of all patients with low (≤8 cm from the anorectal junction [ARJ]), cT3-4 stage rectal cancer were re-reviewed by the participating consultant radiologist in each center after dedicated training. Additionally, MRI scans of all patients with a registered surgical procedure for LLNs, but with a primary tumor >8 cm from the ARJ and/or cT1/2, were also re-reviewed.

### Radiology Re-Review

Two expert radiologists (KH and RBT, with 17 and 24 years’ experience, respectively) provided a 2-h online training session for all participating radiologists, prior to the start of the study. During this session, various examples of LLNs were discussed, as well as the anatomical definitions for the borders of the lateral compartments. The internal iliac compartment contains the lymphatic tissue situated medially of the lateral border of the main trunk of the internal iliac artery. All lymphatic tissue located laterally of the main trunk, and tissue remaining in the lateral compartments after the internal iliac artery exits the pelvis, is considered part of the obturator compartment. External iliac LLNs were defined as those located ventral of the external iliac vessels. Participating radiologists received two color atlases visually portraying these borders, one by Ogura et al.^6^ and one by the Snapshot team, of a complete axial T2-weighted MRI. Both atlases were available during re-review.

Primary and restaging MR images of all patients were re-reviewed by the participating radiologists. The presence, short-axis size, and location of LLNs, along with possible malignant features (heterogeneity, irregular border, round shape, loss of fatty center) were reported. In the case of LR, relevant images were also re-reviewed. An LR was defined as any return of disease situated in the pelvis. An ipsi-lateral LR (LLR) relates specifically to an LR located in a lateral compartment (internal iliac or obturator) on the same side as the enlarged LLN, identified by the reviewing radiologist, and, when applicable, the side that underwent additional LLN surgery.

### Ethics

The Medical Ethics Board of Amsterdam UMC in the Netherlands provided central approval of this study on 30 June 2020. Each center received local approval before participating. Local review boards decided whether their patients were required to provide written informed consent or were given the opportunity to opt-out.

### Statistical Analysis

Statistical analysis was conducted using SPSS Statistics version 26.0 (IBM Corporation, Armonk, NY, USA). Baseline data were evaluated using descriptive statistics. Continuous data are presented as means and standard deviations or medians and interquartile range (IQR), while categorical data are presented as numbers with percentages. Comparative analyses were performed using the Chi-square, Fisher’s exact or independent *t*-tests, as appropriate. Univariable analysis was performed to examine predictors of oncological outcomes. Selected variables included LLN size, anatomical location, and type of LLN surgery. Four-year LR and LLR rates, distant metastasis-free survival, and overall survival were determined using Kaplan–Meier analysis with the log-rank test for comparison.

Multivariable Cox regression models were made to determine predictors of recurrence in patients with primary enlarged LLNs, including whether or not LLN surgery was performed as a variable. Propensity score matching was not possible due to >15% fall-out of cases, causing the group to become unacceptably small. Variables identified during univariable analysis (*p* < 0.1) were included in multivariable analyses. There were too few events for the subsets of clinical T stage in relation to LR and LLR, and they could therefore not be included in the final multivariable analysis model. Statistical significance was set at a *p*-value of ≤0.05.

## Results

**Fig. 1:**
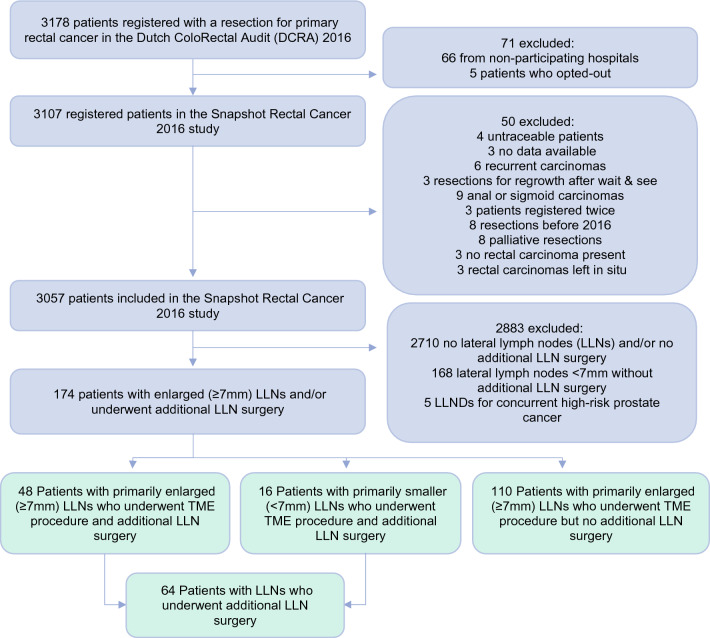
Selection process of included patients. *LLN* lateral lymph node, *LLND* LLN dissection, *TME* total mesorectal excision

In total, 67/69 (97%) Dutch hospitals who performed rectal cancer surgery in 2016 participated, resulting in 3107/3178 (98%) eligible patients (Fig. 1). Of the 3057 patients included in the Snapshot study, 158 had enlarged (≥7 mm short-axis) LLNs, of whom 48 (30%) underwent TME surgery and additional LLN surgery and 110 (70%) received TME surgery only. Another 16 patients also underwent additional LLN surgery, but for LLNs <7 mm only. The 64 patients who underwent additional LLN surgery came from 28 different Dutch hospitals. Five other patients also underwent an LLND but this was performed by the urologist due to synchronous, high-risk prostate cancer, and these patients were therefore not included in this study (Fig. 1). Median follow-up time was 46 months (IQR 18–53 months). Baseline characteristics are displayed in Table 1.

**Table 1:** Baseline characteristics of the 64 patients who underwent additional LLN surgery in 2016

Variable	
Male	38 (59.4)
ASA classification 1–2^a^	51 (79.7)
Age (mean [SD])	62 [10.8]
Lower border of tumor on/below LOREC criteria^b^	38 (59.4)
Clinical T-stage	
T2	7 (10.9)
T3a-d	36 (56.3)
T4a	6 (9.4)
T4b	15 (23.4)
Mesorectal clinical N-stage	
N0	4 (6.3)
N1	23 (35.9)
N2	37 (57.8)
Positive mesorectal fascia	37 (57.8)
Extramural venous invasion (mrEMVI) present on primary MRI	27 (42.2)
Tumor deposits present on primary MRI	12 (18.8)
Short-axis size of LLN present on primary MRI, mm	
≥7	48 (75.0)
<7	16 (25.0)
Anatomical compartment of LLN according to MRI re-review	
Internal iliac	20 (31.3)
Obturator	44 (68.8)
Neoadjuvant treatment	
None	2 (3.1)
Short-course radiotherapy	15 (23.4)
Chemoradiotherapy	46 (71.9)
Monotherapy chemotherapy	1 (1.6)
Operation^c^	
Sphincter-sparing	30 (46.9)
Non-sphincter-sparing	34 (53.1)
Resection margins	
R0	54 (84.4)
R1	10 (15.6)

Data are expressed as *n* (%) unless otherwise specified

*ASA* American Society of Anesthesiologists, *LLN* lateral lymph node, *LOREC* low rectal cancer development program, *MRI* magnetic resonance imaging, *SD* standard deviation

^a^ASA classification score based on physical status

^b^Lower border of the tumor is located beneath the attachment of the levator ani (coronal plane)

^c^Sphincter non-sparing denotes abdominoperineal resections and proctocolectomy cases, and sphincter-sparing includes (low) anterior resections and local excisions

### Lateral Lymph Node Surgery

After central review of surgical reports, LLN surgery was classified as node-picking in 52/64 cases (81%). In 29/52 cases (56%) the report also stated the location of this suspicious node, of which the majority were obturator nodes (23/29, 79%). In the remaining 23 cases (44%), the location was not stated. For 11/29 cases (38%) where a location was stated, the location of the surgical report was discordant with the MRI re-review.

LLN surgery was classified as PRND in the remaining 12 cases (19%). The area of dissection was only the obturator compartment in seven patients, part of the obturator and internal iliac area in two cases, the obturator and external iliac area in two cases, and only the internal iliac area in the remaining patient. There were no discrepancies in location between surgical reports and MRI re-review. None of the surgical reports described which anatomical borders were followed. For the two cases where the obturator and external iliac area were removed together, it was stated that a urologist joined the procedure.

None of the described LLN procedures could be classified as a formal LLND. More details regarding the LLN surgery that was performed are displayed in Table 2.

**Table 2:** Baseline characteristics of the 64 patients who underwent additional LLN surgery in 2016 categorized into node-picking (*n* = 52) and area removal (*n* = 12)

Variable	Node-picking [*n* = 52]	PRND [*n* = 12]	*p*-Value^d^
Male	32 (61.5)	6 (50.0)	0.525
ASA 1–2^a^	42 (80.8)	9 (75.0)	0.697
Age (mean [SD])	62 [10.6]	61 [12.0]	0.888
Clinical T-stage			0.245
T2	7 (13.5)	0	
T3	27 (51.9)	9 (75.0)	
T4	18 (34.6)	3 (25.0)	
Clinical mesorectal N-stage			0.253
N0	2 (3.8)	2 (16.7)	
N1	19 (36.5)	4 (33.3)	
N2	31 (59.6)	6 (50.0)	
Mesorectal fascia positive on primary MRI	29 (55.8)	8 (66.7)	0.491
Extramural venous invasion on primary MRI	22 (42.3)	5 (41.7)	0.968
Tumor deposits on primary MRI	9 (18.0)	3 (25.0)	0.582
Mean short-axis size of LLN, in mm [SD]	9.6 [3.9]	8.8 [3.3]	0.484
Compartment of largest LLN^b^			0.085
Internal iliac	19 (36.5)	1 (8.3)	
Obturator	33 (62.5)	11 (91.7)	
Neoadjuvant treatment			0.164
None	2 (3.8)	1 (8.3)	
Short-course radiotherapy	13 (25.0)	2 (16.7)	
Chemoradiotherapy	37 (71.2)	9 (75.0)	
Operation^c^			0.810
Sphincter-sparing	24 (46.2)	6 (50.0)	
Non-sphincter-sparing	28 (53.8)	6 (50.0)	
Resection margins			0.912
R0	44 (84.6)	10 (83.3)	
R1	8 (15.4)	2 (16.7)	

Data are expressed as *n* (%) unless otherwise specified

*ASA* American Society of Anesthesiologists, *LLN* lateral lymph node, *MRI* magnetic resonance imaging, *SD* standard deviation

^a^ASA classification score based on physical status

^b^According to MRI re-review by participating radiologists

^c^Sphincter non-sparing denotes abdominoperineal resections and proctocolectomy cases, and sphincter-sparing includes (low) anterior resections and local excisions

^d^*P*-values calculated for categorical variables using the Chi-square or Fisher’s exact tests, or independent *t*-tests for continuous variables

### Complications

The status of the obturator nerve was not mentioned in the operative report of 42/64 patients (66%). For the remaining 22 patients, the nerve was spared in 17 cases, damaged in 1 case, and deliberately transected in 4 cases. Significant intraoperative bleeding was reported in six cases (9%), with a mean blood loss of 2333 mL (range 1200–5000 mL). One patient required multiple blood transfusions and admission to the intensive care unit (ICU). In total, 21 (33%) patients required some type of radiological or surgical re-intervention, and 19 (30%) patients were re-admitted at least once.

### Pathology

In 51/64 (80%) cases, LLNs were described separately in the pathology reports. Pathologically positive LLNs were found in 15 (29%) of those patients. The node-positivity rate was 17% (2/12) in patients who underwent PRND and 25% (13/52 patients) after node-picking. Of the 15 pathologically positive LLNs, 4/15 (27%) had a discordant location between surgical report and MRI re-review, and 11/15 (73%) were congruent (*p* = 0.860). All 48 patients with enlarged (≥7 mm) LLNs described LLNs separately in the pathology reports (100%), and 12 were pathologically positive (25%). Node-positivity was 25% for both node picking (10/40) and PRND (2/8).

### Recurrence Rates

In total, nine LLRs occurred after LLN surgery. The primary short-axis diameter of the ipsilateral LN was ≥7 mm in all cases, with a mean size of 10.5 mm. LLR was located in the obturator compartment in six patients and node-picking was performed in eight patients. Excised LLNs harbored metastases in two cases (Appendix 2).

For 64 patients who underwent LLN surgery, the 4-year LR and ipsi-lateral LR rates were 26% and 15%, respectively. When examined according to technique, the 4-year LR rate was 22% for node-picking and 46% after PRND (*p* = 0.104). Corresponding 4-year LLR rates were 16% and 9%, respectively (*p* = 0.582). LLR rates were not significantly different for the 44 patients who underwent a restaging MRI and had LLNs that disappeared, shrunk, or grew on the restaging MRI after neoadjuvant treatment (0%, 15.4%, 20.0%, respectively; *p* = 0.293). For these 64 patients who underwent additional LLN surgery, the 4-year distant metastasis-free and overall survival rates were 58.1% and 58.4%, respectively.

Forty-eight patients who underwent LLN surgery (75%) had at least one LLN with a short-axis diameter ≥7 mm. A total of 110 patients with enlarged internal iliac and/or obturator LLNs underwent TME surgery only. The baseline characteristics of patients with enlarged LLNs who underwent TME surgery with or without LLN surgery are displayed in Table 3. In the TME-alone group, a higher proportion of patients had cT3 stage, a lower proportion had N2 stage, and the mean short-axis diameter was slightly smaller (9.2 vs. 10.8 mm). Otherwise, the groups were comparable. The 4-year LR rate was 26% for patients who underwent additional LLN surgery, compared with 20% for those without additional surgery (*p* = 0.256). The 4-year LLR rates were 19% and 13%, respectively (*p* = 0.138). Multivariable analysis did not reveal a significant association between type of surgical treatment and LR (Table 4) or LLR (Table 5). Additional LLN surgery resulted in a hazard ratio (HR) >1 (LR: HR 1.533, 95% confidence interval [CI] 0.724–3.244, *p* = 0.264; LLR: HR 1.924, 95% CI 0.247–2.463, *p* = 0.874) [Tables 4 and 5]. A subanalysis of the same patient groups, but only for patients with tumors ≤4 cm from the ARJ, revealed similar results, with 4-year LR and LLR rates of 34% versus 26% (*p* = 0.518) and 18% versus 14% (*p* = 0.700), respectively (Appendix 3).

**Table 3:** Patients with lateral lymph nodes with short-axis ≥7 mm who did (*n* = 48) or did not (*n* = 110) undergo additional LLN surgery (area removal or node-picking) for their enlarged lateral lymph node

Variable	LLN surgery [*n* = 48]	No LLN surgery [*n* = 110]	*p*-Value^d^
Male	33 (68.8)	73 (66.4)	0.769
ASA 1–2^a^	36 (75.0)	93 (84.5)	0.154
Age (mean [SD])	61.4 [10.6]	64.0 [11.7]	0.185
Distance of tumor from the anorectal junction			0.644
Low (0–4 cm)	35 (72.9)	84 (76.4)	
High	13 (27.1)	26 (23.6)	
Clinical T-stage			< 0.001
T2	6 (12.5)	0	
T3	26 (54.2)	80 (72.7)	
T4	16 (33.3)	30 (27.3)	
Mesorectal cN-stage			0.017
N0	0	16 (14.5)	
N1	18 (37.5)	40 (36.4)	
N2	30 (62.5)	54 (49.1)	
Mesorectal fascia positive on primary MRI	29 (60.4)	65 (59.1)	0.876
Extramural venous invasion on primary MRI	25 (52.1)	40 (36.4)	0.065
Tumor deposits present on primary MRI	11 (22.9)	15 (13.6)	0.148
Mean short-axis size of LLN, in mm [SD]	10.8 [3.3]	9.2 [2.9]	0.006
Compartment of largest LLN^b^			0.523
Internal iliac	14 (30.4)	28 (25.5)	
Obturator	32 (69.6)	82 (74.5)	
Neoadjuvant treatment			0.611
None	2 (4.2)	7 (6.4)	
Short-course	10 (20.8)	29 (26.4)	
Chemoradiotherapy	36 (75.0)	74 (67.3)	
Operation^c^			0.818
Sphincter-sparing	20 (41.7)	48 (43.6)	
Non-sphincter-sparing	28 (58.3)	62 (56.4)	
Resection margins			0.752
R0	41 (85.4)	96 (87.3)	
R1	7 (14.6)	14 (12.7)	

Data are expressed as *n* (%) unless otherwise specified

*ASA* American Society of Anesthesiologists, *LLN* lateral lymph node, *MRI* magnetic resonance imaging, *SD* standard deviation

^a^ASA classification score based on physical status

^b^According to MRI re-review by participating radiologists

^c^Sphincter non-sparing denotes abdominoperineal resections and proctocolectomy cases, and sphincter-sparing includes (low) anterior resections and local excisions

^d^*P*-values calculated for categorical variables using the Chi-square or Fisher’s exact tests, or independent *t*-tests for continuous variables

**Table 4:** Multivariable regression analysis of *local recurrence* in 158 patients with lateral lymph nodes with short-axis diameter ≥7.0 mm who either did (*n* = 48) or did not (*n* = 110) undergo additional surgical treatment for these lateral lymph nodes

Variable	*N*	Univariate analysis			Multivariate analysis		
		HR	95% CI	*p*-Value	HR	95% CI	*p*-Value
Surgical procedure for LLN				0.260			0.264
No	110	1			1		
Yes	48	1.509	0.737–3.087		1.533	0.724–3.244	
Mesorectal clinical N stage				0.490			
N0	16	1					
N1	58	1.006	0.272–3.716				
N2	84	1.553	0.461–5.231				
Extramural venous invasion				**0.031**			0.054
Present	65	2.161	1.074–4.346		2.107	0.986–4.502	
Tumor deposits				**0.012**			0.053
Present	26	2.798	1.254–6.247		2.467	0.990–6.152	
Neoadjuvant radiotherapy				**0.006**			**<0.000**
None	9	1			1		
Short-course radiotherapy	39	0.296	0.099–0.882		0.143	0.042–0.490	
Chemoradiotherapy	110	0.296	0.090–0.56		0.134	0.050–0.355	
Margin status				**0.032**			**0.011**
R0	137	1			1		
R1	21	2.517	1.083–5.848		3.317	1.309–8.404	

*HR* hazard ratio, *CI* confidence interval, *LLN* lateral lymph node

**Table 5:** Multivariable regression analysis of *lateral local recurrence* in 158 patients with lateral lymph nodes with short-axis diameter ≥7.0 mm who either did (*n* = 48) or did not (*n* = 110) undergo additional surgical treatment for these lateral lymph nodes

Variable	*N*	Univariate analysis			Multivariate analysis		
		HR	95% CI	*p*-Value	HR	95% CI	*p*-Value
Surgical procedure for LLN				0.145			0.874
No	110	1			1		
Yes	48	1.901	0.801–4.512		1.924	0.247–2.463	
Mesorectal clinical N stage							
N0	16	1		0.402			
N1	58	0.830	0.161–4.282				
N2	84	1.622	0.368–7.144				
Extramural venous invasion				**0.001**			**0.010**
Present	65	5.348	1.959–14.600		3.992	1.393–11.444	
Tumor deposits				**0.015**			0.062
Present	26	3.249	1.255–8.413		2.590	0.952–7.047	
Neoadjuvant radiotherapy				0.211			
None	9	1					
Short-course radiotherapy	39	0.884	0.183–4.266				
Chemoradiotherapy	110	0.418	0.093–1.868				
Margin status				0.193			
R0	137	1					
R1	21	2.067	0.692–6.172				

*HR* hazard ratio, *CI* confidence interval, *LLN* lateral lymph node

The 48 patients with LLNs ≥7 mm who underwent LLN surgery had significantly lower distant metastasis-free survival (56.9% vs. 64.6%, *p* = 0.044), but statistically similar overall survival rates (59.8% vs. 74.4%, *p* = 0.141) compared with the 110 patients with enlarged LLNs who did not undergo LLN surgery. Overall survival rates dropped in the non-LLN surgery group after 4 years, to reach similar levels as the LLN surgery group, which explains the *p*-value.

## Discussion

The current study provides insights into LLN procedures during current daily practice for rectal cancer in the Netherlands. Some form of LLN surgery was performed in 2% of the patients in 28 Dutch centers. Of all patients with primary enlarged LLNs, one-third underwent LLN surgery, mainly consisting of node-picking. The LLR rate after node picking/PRND in patients with primary enlarged LLNs was 19%, and 13% in similar patients who underwent TME surgery alone. HRs >1 suggested that incomplete LLN surgery was associated with a higher risk of (L)LR in multivariable analyses, although not reaching statistical significance. Considering that multiple studies show an LLND significantly lowers the LLR risk to rates of around 6%,^5–7,17,18^ the current results indicate that LLN surgery in an untrained setting consisting of node-picking or PRND does not result in adequate local control.

There are multiple possibilities as to why the LLR rate was high after LLN surgery in this cohort. If a formal complete LLND is not performed, tissue with micrometastases can be left behind, which later develops into a recurrence. None of the operative reports described whether specific anatomical borders were followed, insinuating that likely not all tissue from the lateral compartments was removed. One study of 66 patients who underwent LLND, with a total of 892 examined LLNs, found positive cytokeratin-staining for micrometastases of initially negative LLNs in 19% of patients.^19^ These patients had similar survival outcomes compared with those with positive LLNs, and significantly worse outcomes than the patients without positive LLNs. A similar study of 67 patients with 726 examined LLNs found that 10 patients with micrometastases and 12 patients with positive LLNs had similarly high recurrence and poor survival outcomes, compared with the 45 patients with negative LLNs (LR: 33%, 30%, and 6.7%, respectively; *p* < 0.001).^19^ Another possibility is that dissection of a single node, or a few nodes, may have caused tumor spill because such dissections do not follow oncological principles by not respecting anatomical planes and potentially compromising margins around grossly involved nodes.^20^

Alternatively, it is possible that the wrong areas were removed. For example, in only two cases, the obturator and internal iliac areas were supposedly removed, while in the remaining 10 cases, other areas such as the obturator and external iliac were removed, or only the obturator compartment was removed. Previous studies show that obturator and internal iliac LLNs are the most anatomically and clinically relevant for rectal cancer patients with LLN metastases.^6,7,21,22^ Removal of the obturator and external iliac areas together is likely not satisfactory in rectal cancer cases, in contrast to prostate cancers for which these areas are the primary lymphatic drainage areas belonging to the primary tumor site. This is why an LLND in rectal cancer cases should be performed by a colorectal surgeon and not an urologist.^1^ Furthermore, eight of the nine patients who developed an LLR (Appendix 2) underwent node-picking. Considering the development of LLR in these patients, it is possible that the wrong LLN was removed. Only two other studies with very small samples investigated node-picking and reported LLR rates ranging up to 51%.^7,15^ This again suggests that node-picking is insufficient in procuring lower LLR rates. In fact, an overall trend towards higher LR and LLR rates was found when performing additional LLN surgery, compared with patients with enlarged LLNs who did not. We were able to define a control group with TME surgery alone, and the unfavorable LR and LLR rates support the hypothesis that untrained incomplete LLN surgery might have even worsened the outcomes. The lack of standardization and consensus regarding the (surgical) treatment of LLNs is problematic and needs to be addressed by introducing appropriate training.

Another issue is the low rate of pathologically positive LLNs. Earlier research indicated that after complete LLND, pathological positivity rates range up to 75%.^6^ In that case, the 20% positivity rate found here is low. One important point is that the Consortium study was performed in expert centers only, with most likely high exposure of surgeons and pathologists to the LLND procedure and specimens. Pathologists in these centers may therefore have more knowledge and awareness to investigate and report them separately compared with the current untrained setting. Another possibility is that an ‘incorrect’ node or area was removed. In that case, other malignant LLNs were left behind to potentially cause an LLR. In a previous node-picking study, it was found that the tissue removed did not always contain an LLN,^15^ explaining the occurrence of pathological negativity.

It is important to realize that in this study there might be some selection bias. It is possible that in 2016, patients with more aggressive tumors underwent LLN surgery more often, which may be reflected by the lower distant metastasis-free survival in these patients. However, results from a larger cohort (1109 patients with low [≤8 cm], locally advanced [cT3+] tumors) from this same Snapshot study found that only one-third of patients metastasized during the 4-year follow-up period, and overall survival was not significantly different for patients with enlarged LLNs versus those without.^21^ This would suggest that disease advancement is not solely or directly related to lateral node status. Similarly, no statistically significant difference was found for the current cohort in terms of overall survival for patients with versus without LLNs. However, even if the patients who underwent LLN surgery had more aggressive tumors, a proper LLND would have to prevent LLR and this risk rate should be around 6%, as published in previous literature^5–7,17,18^

Ultimately, the surgical techniques used in 2016 for these 64 patients with LLNs did not seem sufficient to prevent lateral nodal recurrences. These rates may be influenced by the increase in total neoadjuvant therapy (TNT) with systemic chemotherapy, combined with CRT, which has demonstrated promising effects on LR rates. However, surgical training and standardization may improve rates further, as many trials investigating LLND demonstrate better long-term LLR results. A number of published reports discuss the removal of lateral lymphatic tissue according to standardized anatomical borders. This would ensure that all tissue, including areas with possible micrometastases, can be removed and reduces the chances of tumor spill or micrometastases being left behind. LLND following anatomical borders via training with a sufficient learning curve may improve these rates. Similar surgical training programs have been created for other niche operations^23^ with positive results. LLNDs performed in the prospective LaNoReC study are only performed by trained surgeons, which hopefully will result in lower LR rates.

There are several limitations to this study, primarily the retrospective design with only operative reports and without surgical videos, and the limited number of LLN procedures. Furthermore, the operations were performed in 2016, and hence the situation may already be very different in the present day. It is also possible that the anatomical location according to the operation report is not wholly trustworthy, as exact consensus for the definitions for the borders of the compartments was lacking in 2016, making variation very possible. There was also no re-review of the pathology outcomes, only details from the reports. Furthermore, the current cohort may reflect a patient selection with more aggressive cancer biology, potentially contributing to the higher (L)LR outcomes. However, in the literature, even in these more advanced cancers, acceptable LLR rates can be achieved with formal LLND.

## Conclusion

In a Dutch rectal cancer population treated in 2016, only 2% of patients underwent some form of additional LLN surgery, mainly consisting of node-picking. Patients with primarily enlarged (≥7 mm) LLNs who underwent TME with additional LLN surgery had a 4-year LLR rate of 19%, while in similar patients who underwent TME only, this rate was 13%. Previous literature suggests that a formal LLND is able to improve long-term oncological outcomes and this should be investigated after thorough training.

## Data Availability

Data, analytic methods, and study materials can be made available upon reasonable request to the corresponding author.

## Appendix 1: Data management

Data were stored and processed anonymously by Medical Research Data Management (MRDM, Deventer, the Netherlands). MRDM is also responsible for the management of the Dutch Colorectal audit (DCRA) data and is NEN7510 and ISO27001 certified. The DCRA holds information regarding all patients operated on for colorectal cancer in the Netherlands and includes short-term oncological follow-up outcomes.

The current Snapshot study collected data from each participating centre who gathered the data of their own patients. Data collection was separated into three parts and the data was also restricted within each section so that each specialist only had access to their own data and patients. Isolated data extracts were performed once each part was complete. Patients eligible for multiple parts had a study number in all three sections so that their data could be combined at a later stage.

Only one-way, fully anonymized, data was sent to the central coordinating researchers. One-way configuration means that it was impossible to retrace/decode information at a later stage once the data was coded. Dates of birth were only provided as a year of birth which all other dates were given a possible ten day spread, to minimize any breaches in privacy of traceability. Any provided reports were copied by the local collaborative team into the database anonymously and were checked by MRDM to ensure that no patient-specific information was included before being included in the final dataset.

## Appendix 2: Case descriptions of patients who developed an ipsi-lateral local recurrence during follow-up period

**Table:**

	Gender	Age	cT stage	cN stage	LLN size according to MRI re-review	Location according to surgical report & MRI re-review	EMVI present	Tumour deposits present	MRF positive	Type of neoadjuvant treatment	Restaging LLN size	Primary operation	Resection margins	pT stage	pN stage	LLN PA+*
**Case 1**	Female	<55	3	2	16.4mm	Obturator	Yes	Yes	Yes	CRT (25x2Gy)	17.5mm	SNS	R0	3	2	Yes
**Case 2**	Female	55-75	3	1	12.0mm	Internal iliac	No	No	No	None	12.0mm	SS	R0	3	2	No
**Case 3**	Male	55-75	4	1	10.7mm	Internal iliac	Yes	Yes	Yes	CRT (28x1.8Gy)	7.6mm	SNS	R1	4	1	No
**Case 4**	Male	>75	3	1	9.0mm	Internal iliac	No	No	Yes	CRT (28x1.8Gy)	9.0mm	SS	R0	0	2	No
**Case 5**	Male	>75	3	1	9.7mm	Obturator	No	No	Yes	5x5 Gy	4.1mm	SNS	R0	0	0	No
**Case 6**	Male	55-75	3	2	7.8mm	Obturator	Yes	No	No	CRT (25x2 Gy + boost to 60Gy on LLN)	9.2mm	SS	R0	3	2	Yes
**Case 7**	Female	<55	4	2	8.0mm	Obturator	Yes	No	Yes	CRT (25x2Gy)	8.0mm	SS	R0	4	0	No
**Case 8**	Male	<55	3	2	10.0mm	Obturator	Yes	Yes	Yes	5x5 Gy	4.0mm	SS	R0	3	2	No
**Case 9**	Male	55-75	4	2	11.0mm	Obturator	Yes	Yes	No	5x5 Gy	6.0mm	SNS	R0	3	1	No

CRT: chemoradiotherapy, SCRT: short-course radiotherapy, SNS: sphincter-non sparing (abdominoperineal resection or proctocolectomy), SS: sphincter-sparing (low anterior resection/total mesorectal excision). *PA+: positive LLN found during pathology analysis.

## Appendix 3: Sub-analysis according to tumor location

When considering the patients with enlarged (≥7mm) lateral lymph nodes who did undergo additional LLN surgery or did not, but were also located ≤4cm of the anorectal junction, this results in a total of 119 patients; 35 underwent LLN surgery and 84 did not.

For these 119 patients, there were no significant differences in oncological survival outcomes for the 35 patients who underwent additional LLN surgery compared to the 84 patients who did not. Four-year overall survival was 52.8% versus 71.8% (p=.700) and distant metastasis free survival was 56.9% versus 62.5% (p=.074), respectively.

Local recurrence rates were as follows: local recurrence occurred in 26.3% of patients undergoing additional LLN surgery compared to 34.1% without LLN surgery (p=.518), while lateral local recurrence rates were 14.1% versus 18.8% (p=.700), respectively.

**Table:**

Variable (only ≤4cm from anorectal junction)	LLN surgery N (%) (n=35)	No LLN surgery N% (n=84)	*p*-Value
Male	29 (82.9)	52 (61.9)	.025
ASA^a^: 1-2	28 (80.0)	69 (82.1)	.784
Age (mean, SD)	67 (10.9)	69 (11.4)	.309
Clinical T-stage			.001
T2	5 (14.3)	0	
T3	17 (48.6)	61 (72.6)	
T4	13 (37.1)	23 (27.4)	
Mesorectal cN-stage			.031
N0	0	12 (14.3)	
N1	11 (31.4)	31 (36.9)	
N2	24 (68.6)	41 (48.8)	
Mesorectal fascia positive on primary MRI	22 (73.3)	53 (63.1)	.112
Extramural venous invasion on primary MRI	17 (50.0)	30 (35.7)	.151
Tumour deposits present on primary MRI	8 (23.5)	8 (9.5)	.071
Mean short-axis size of LLN in mm (SD)	11.0 (3.3)	9.4 (3.1)	.013
Compartment of largest LLN^b^			602
Internal iliac	11 (31.4)	19 (22.6)	
Obturator	24 (68.6)	65 (77.4)	
Neoadjuvant treatment			.104
None	0	7 (8.3)	
Short-course or chemoradiotherapy	35 (100l.0)	77 (91.7)	
Operation^c^			.451
Sphincter-sparing	12 (34.3)	23 (27.4)	
Non-sphincter sparing	23 (65.7)	61 (72.6)	
Resection margins			.438
R0	28 (80.0)	72 (85.7)	
R1	7 (20.0)	12 (14.3)	

^a^ASA classification score based on physical status

^b^According to MRI re-review by participating radiologists

^c^Sphincter non-sparing denotes abdominoperineal resections and proctocolectomy cases, and sphincter-sparing includes (low) anterior resections and local excisions
